# Identification of a New Genomic Hot Spot of Evolutionary Diversification of Protein Function

**DOI:** 10.1371/journal.pone.0125413

**Published:** 2015-05-08

**Authors:** Aline Winkelmann, Xiantian You, Nora Grünewald, Ute Häussler, Heinz Krestel, Carola A. Haas, Günter Schwarz, Wei Chen, Jochen C. Meier

**Affiliations:** 1 RNA editing and Hyperexcitability Disorders Group, Max Delbrück Center for Molecular Medicine, Berlin, Germany; 2 Laboratory of Functional and Medical Genomics, Max Delbrück Center for Molecular Medicine, Berlin, Germany; 3 Department of Biochemistry, University of Cologne and Center for Molecular Medicine, Cologne, Germany; 4 Department of Neurosurgery, University of Freiburg, Freiburg, Germany; 5 Department of Neurology, Bern University Hospital, Bern, Switzerland; 6 Life Science Department, Zoological Institute, Division of Cell Physiology, TU Braunschweig, Braunschweig, Germany; International Centre for Genetic Engineering and Biotechnology, ITALY

## Abstract

Establishment of phylogenetic relationships remains a challenging task because it is based on computational analysis of genomic hot spots that display species-specific sequence variations. Here, we identify a species-specific thymine-to-guanine sequence variation in the *Glrb* gene which gives rise to species-specific splice donor sites in the *Glrb* genes of mouse and bushbaby. The resulting splice insert in the receptor for the inhibitory neurotransmitter glycine (GlyR) conveys synaptic receptor clustering and specific association with a particular synaptic plasticity-related splice variant of the postsynaptic scaffold protein gephyrin. This study identifies a new genomic hot spot which contributes to phylogenetic diversification of protein function and advances our understanding of phylogenetic relationships.

## Introduction

The phylogenetic tree of *Eutherian* (placental mammal) evolution is not yet established but analyses of phylogenetic relationships are ongoing, rely mostly on morphological, behavioral and genetic parameters and consult identified genome regions with significant species-wide genome variations. We recently discovered an unexpected PCR amplification product of the neurotransmitter receptor for glycine (GlyR) in mouse cells [1]. The region of interest corresponds to the large cytosolic loop between transmembrane domains 3 and 4 of the GlyR β subunit which is a relevant protein domain involved in postsynaptic GlyR clustering.

Neuronal communication involves synaptic transmission, and glycine-/GABA-dependent inhibition plays an important role as it not only counterbalances excitation but also provides a spatiotemporal framework for behaviorally relevant neuronal integration of synaptic signals [2–4]. Alternative RNA splicing diversifies the function of proteins involved in synaptic inhibition; we showed that the postsynaptic GlyR and GABA type A receptor (GABA_A_R) anchoring protein gephyrin [5–10] undergoes extensive alternative RNA splicing which contributes to cell type-specific expression of gephyrin splice variants [11] and regulation of postsynaptic glycine receptor (GlyR) clustering [12,13]. On the other hand, GlyRs and GABA_A_Rs also undergo extensive alternative RNA splicing which regulates receptor clustering [14–17] and subcellular receptor localization [3].

Here, we present a new species-specific RNA splice variant of the GlyR β subunit and identify the *Glrb* gene as a new genomic hot spot of phylogenetic diversification of protein function.

## Materials and Methods

### Molecular cloning

The GlyR HA-α1-βE9A chimera was generated using Fusion-PCR. For the insertional mutagenesis, oligonucleotides 5’-GGCTGGCCAACAGACACGCTCACCACACAGAACCCCGCTCCTGCACCGTC-3’ and 5’- TGTTGGCCAGCCATCCAGTTGGTAAGTGATCGTGGTGTTGTTGTTGTTGGCAC-3’ were used and the resulting PCR product was digested using MscI restriction enzyme which recognizes the TGGCCA sequence present in the middle of the E9A-3 sequence. For this purpose, the GlyR HA-α1 construct [7] was used as PCR template. Thus, the sequence ITYQLDGWPTDTLTTQ was inserted following the NNNNTT sequence of GlyR α1, at the position corresponding to the validated chimeric GlyR α1-βgb construct [5]. GST-GlyR β large cytosolic loop constructs were generated using PCR with oligonucleotides 5’-gtatgccgaattccaggtgatgttgaacaa-3’ and 5’- GAACAAGAAGCACTCGAGTTATAATGCTCTTGC-3’ followed by EcoRI and XhoI restriction digest. The fragments were cloned into the pGEX-6P-1 expression vector (GE Healthcare Life Sciences, USA). The GlyR βTM3-4 loop domains (QVMLN…YARAL) contain the βgb peptide RSNDFSIVGSLPRDFELS, βgb and E9A-3 (ITYQLDGWPTDTLTTQ), or E9A-3 alone. For the latter construct, Fusion PCR with oligonucleotides 5’-TGAAAGATCTAATTATGACTGCTATGGG-3’ and 5’-CATTAGATCTCAGATCAGACTTGG-3’ and *Bgl*II restriction digest were used, leaving the AGATCT-encoded sequence RS of the βgb sequence. Cloning of EGFP-tagged-gephyrin splice variants was described previously [11].

### Cell culture and transfection

Spinal cord cultures from embryonic day 14 (E14) were prepared as described [18], and primary hippocampal neurons were obtained likewise, except that E18 embryos were used [12]. Cultures were maintained in B27- and 1% FCS-supplemented Neurobasal medium [19]. Initial cell plating density was 68,000/cm^2^. Transfection and protein expression were carried out on day *in vitro* (DIV) 11. For transfection, coverslips were transferred to wells containing transfection medium (Neurobasal supplemented with 0.25 mM glutamine) and were incubated with complexes formed with 5 μL of Effectene transfection reagent (Qiagen, Hilden, Germany) and 300 ng of DNA. The Qiagen transfection protocol was followed, except that the incubation time was reduced to 1.5 h. This protocol ensured moderate protein expression levels within 3 days in ~1% of primary neurons. The study received institutional approval and experiments were carried out in accordance with the European Communities Council Directive regarding the care and use of animals for experimental procedures (2010/63/EU). In agreement with that, the Ethics Committee of the Office for Health Protection and Technical Safety of the Regional Government of Berlin (Landesamt für Gesundheit und Soziales Berlin) approved this study as all animals were killed according to the permit LaGeSo No. T0122/07.

### Antibodies and microscopy

Immunochemistry was performed as described earlier [7,12,20]. HA-tagged GlyR chimaeras were stained with a rat monoclonal HA antibody (clone 3F10, 1:100; Roche Applied Science, Mannheim, Germany). To identify glycinergic/GABAergic synapses, the vesicular inhibitory amino acid transporter (VIAAT) was visualized using a guinea pig polyclonal antibody (#131 004, 1:300; Synaptic Systems GmbH, Göttingen, Germany), and gephyrin using a polyclonal rabbit antibody (#147 003, 1:100; Synaptic Systems GmbH). For multiple labelling experiments, monoclonal and polyclonal antibodies were combined. Secondary antibodies coupled to Cy3, Cy5, FITC or AMCA were purchased from Jackson ImmunoResearch Laboratories (West Grove, PA, USA). Transfected EGFP-tagged gephyrin constructs were visualized according to EGFP [21] fluorescent signals. Coverslips were mounted in Vectashield medium (Vector Laboratories, Burlingame, CA, USA). Appropriate filters (U-MSP100v2 MFISH DAPI, U-MSP101v1 MFISH FITC, U-MSP102v1 MFISH Cy3 and U-MSP104v1 MFISH Cy5; Olympus GmbH, Germany) allowed the detection and separation of fluorescent signals. To ensure that labelling was specifically due to the primary antibodies, we replaced the latter with similarly diluted normal serum from the same species. Labelled neurons were visualized with a standard epifluorescence microscope (Olympus BX51; Olympus Deutschland GmbH, Hamburg, Germany) under U Plan Apo ×40 oil objective with a numerical aperture of 1.00 (Olympus). Images were acquired using a 14-bit cooled CCD camera (Spot PURSUIT; Visitron Systems GmbH, Puchheim, Germany).

### Quantification of postsynaptic immunofluorescence intensities

Fluorescence intensities were measured using the software Metamorph (Universal Imaging Corp., Downingtown, PA, USA) and line scans of VIAAT-, gephyrin- and HA-GlyR-positive inhibitory synapses were recorded as described [7]. Briefly, fluorescence intensities (255 grey levels) corresponding to synaptic (VIAAT-positive) HA-GlyR and gephyrin signals were extracted, and ratios of pixel intensities between HA-GlyR and gephyrin signals were calculated along 2–3 μm length intervals centered on postsynaptic clusters and used for correlation analysis. To determine congruency of HA and gephyrin signal intensities within postsynaptic GlyR clusters, the same approach and set of data were used except that the length interval was reduced to at least 0.66 μm (4 pixels) so that it covered the HA fluorescence intensity peak. Again, ratios of pixel intensities corresponding to HA and gephyrin signals were calculated. Thus, a value of 1 indicates perfect congruency (overlap) of the HA signal peak and the gephyrin signal within postsynaptic receptor clusters.

### Biochemical analyses

GST-β loop variants were expressed in *E*. *coli* BL21. Cells were grown in LB medium at 37°C until protein expression was induced at OD_600_ = 0.6 with 100 μM isopropyl β-D-1-thiogalactopyranoside and harvested after 4 h of expression at 22°C. Cell lysates were prepared in PBS, containing protease inhibitors, and incubated with glutathione agarose matrix. Bound GST-β loop variants were extensively washed with PBS and stored in PBS containing protease inhibitor. Co-sedimentation experiments were performed with mouse brain lysates as described ^7^. Briefly, brain homogenate was resuspended in 100 mM Tris/HCl pH 8.0, containing protease inhibitors, and cleared twice by centrifugation at 15,000 rpm for 20 min at 4°C. Mouse brain lysate (350 μg) was incubated with equal amounts of GST-β loop-loaded glutathione agarose in a final volume of 200 μl, filled up with PBS. Samples were incubated for 30 min at room temperature under mild shaking, followed by five washing steps with PBS. Gephyrin in the pellet and supernatant fractions was detected following western blotting and incubation with a monoclonal antibody directed against the E-domain of gephyrin (mAb3B11; [11]).

### Sequence analyses

We looked into the publicly available RNA sequence datasets to estimate the relative abundance of GlyR βE9A-3 in different tissues, species and conditions. The following 15 different mouse strains were included: 129S1/SvImJ (ERS028660), A/J (ERS028666), AKR/J (ERS028672), BALB/cJ (ERS028670), C3H/HeJ (ERS028658), C57BL/6NJ (ERS028664), CAST/EiJ (ERS028668), CBA/J (ERS028662), DBA/2J (ERS028659), LP/J (ERS028671), NOD/Ltj (ERS028663), NZO/HlLtJ (ERS028667), PWK/PhJ (ERS028661), Spretus/EiJ (ERS028665) and WSB/EiJ (ERS028669). For expression analysis, we downloaded raw data, aligned the reads to genome references using Tophat2 [22] and counted the number of splicing-junction supporting reads for E9A-2, E9A-3, E9A-4 and the exon skipping events.

### Evolutionary analyses

The evolutionary history of the GlyR βE9A sequences was inferred using the Neighbor-Joining method [23]. The optimal tree with the sum of branch length = 3.42402700 is shown. The evolutionary distances were computed using the Maximum Composite Likelihood method [24] and are in the units of the number of base substitutions per site. The differences in the composition bias among sequences were considered in evolutionary comparisons [25]. The analysis involved sequences from 27 species, the multiple sequence alignment of which was downloaded from the Conservation track in the UCSC Genome Browser (60 vertebrates). All ambiguous positions were removed for each sequence pair. Evolutionary analyses were conducted in MEGA6 [26].

### Statistics

Numerical data are reported as mean ± SD. Statistical analysis (ANOVA followed by post hoc Bonferroni’s test) was performed using the software Origin (Microcal, Northampton, MA, USA). Significance levels derived from post hoc Bonferroni’s analysis are indicated as * (*P* < 0.05) and *** (*P* < 0.001).

## Results

**Fig 1:**
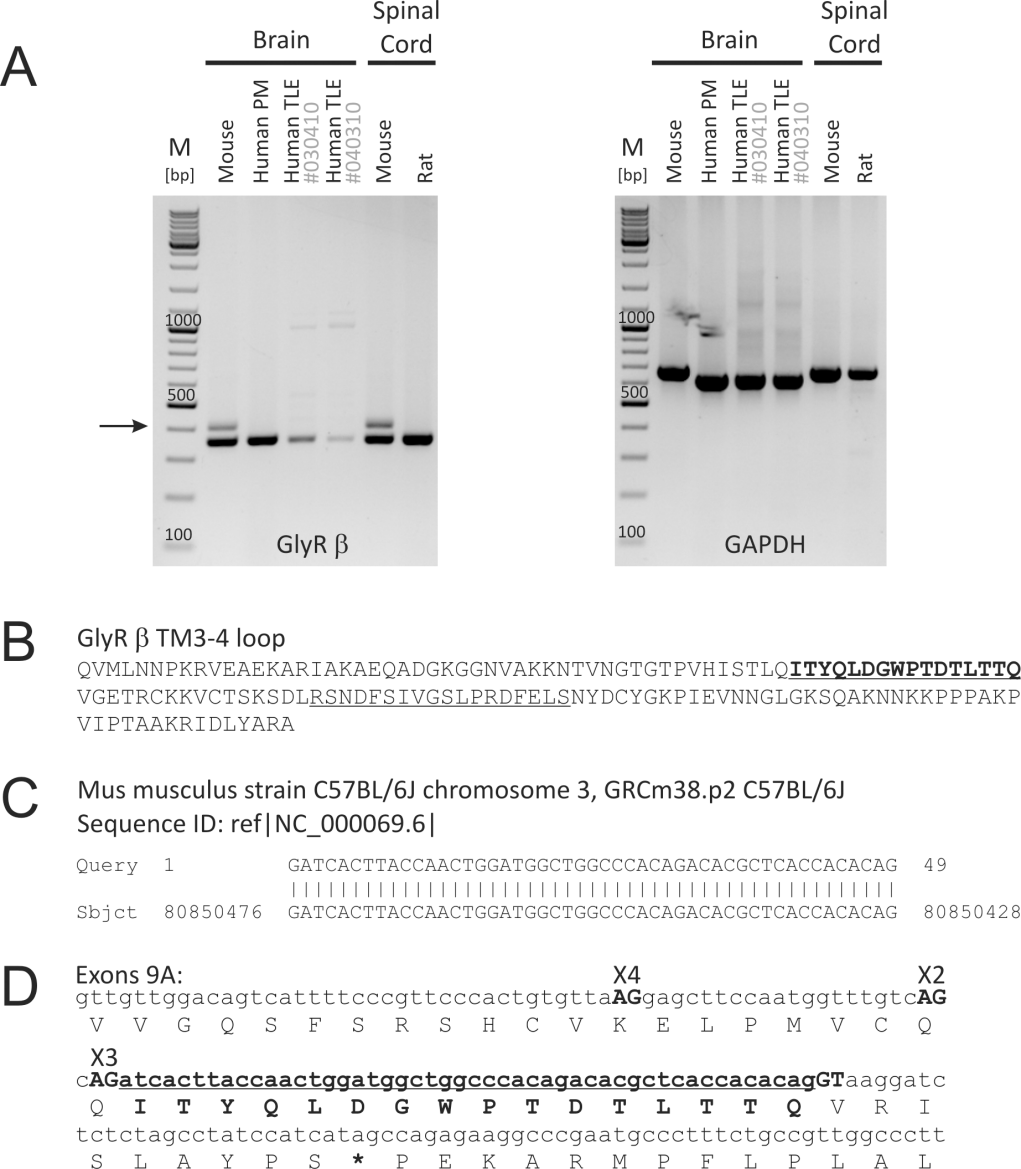
RT-PCR analysis of GlyR β RNA splicing. (A) PCR using oligonucleotides spanning the large cytosolic loop between transmembrane domains 3 and 4 revealed an additional band (arrow). The additional band seems to be specific for mouse cDNA samples (e.g. brain [hippocampus] and spinal cord) as it was not detected in human brain (“Human PM”: human postmortem hippocampus; provided by Clontech “Human TLE #030310”: human cortex from epilepsy patient #030310; “Human TLE #040310”: human cortex from epilepsy patient #040310, see [32] for medical history) or rat spinal cord. (B) Molecular cloning and sequence analysis of the additional PCR band identified a new GlyR β variant which contains 16 additional amino acids (bold and underlined). The new insert is located upstream of the established gephyrin binding sequence (βgb) of the GlyR β subunit (underlined). (C, D) Blast search revealed 100% homology with the depicted mouse genome region on chromosome 3, downstream of exon 9 and upstream of exon 10 (C). Actually, two additional splice acceptor sites (X4 and X2) are located upstream of the splice acceptor site X3 which gives rise to the cloned splice variant (D, sequence in bold and underlined).

We subjected cDNA samples of different brain regions and species to RT-PCR analysis and detected the additional PCR amplification product [1] consistently in mouse samples, not in human or rat samples (Fig 1A, arrow). Densitometric analysis of band intensities revealed that the relative expression level of this additional band compared to the expected regular band was ~20% in all cases including mouse neurological disease models (S1 and S2 Figs). Molecular cloning of the additional band showed that the insert codes for the amino acid sequence ITYQLDGWPTDTLTTQ (Fig 1B). A BLAST search showed that the additional mRNA sequence corresponds to a genomic region on chromosome 3 within the *Glrb* gene in mice (Fig 1C). As the new exon is located downstream of exon 9 of the *Glrb* gene in mice, we name it “exon 9A, E9A” (Fig 1D). The sequence of E9A is found in Muridae (e.g. *Mus musculus*, *Rattus norvegicus*) and Cricetidae (e.g. *Microtus ochrogaster*, *Mesocricetus auratus*, or *Peromyscus maniculatus bairdii*). With some sequence variation, the sequence of E9A is also found in other species including *Homo sapiens* and *Otolemur* (bushbaby) (for alignment of genome sequences see S3 Fig). Interestingly, the splice donor site GT is present only in mouse and bushbaby, not in any other species (S3 Fig). To further elucidate the origin of the new splice donor site, we surveyed the sequenced mouse genomes reported in [27], which include a representative of *Mus spretus*, the sister species to *Mus musculus*. We found that all sequenced mouse strains include the new splice donor site, implying that its origin predates the split between *Mus spretus* and *Mus musculus* ~1.5 million years ago. The fact that the putative splice donor site in *Otolemur* has a predicted splicing strength value of 6.33, according to a computational analysis using the maximum entropy modeling of short sequence motifs [28], suggests that the splice donor site may be functional in bushbaby and may actually have evolved already 40–50 million years ago.

**Fig 2:**
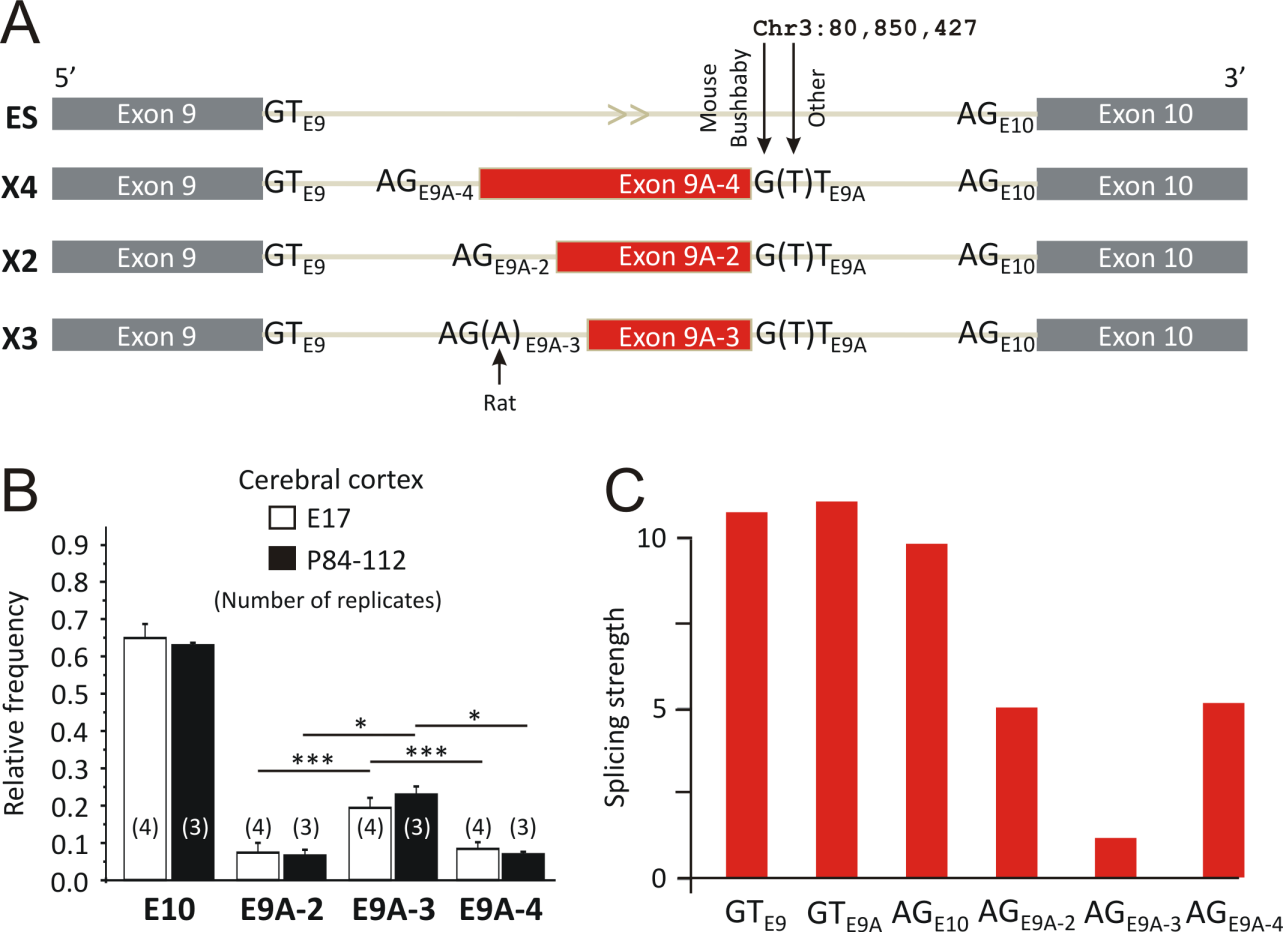
Species-specific alternative RNA splicing of GlyR β exon 9A. (A) The scheme illustrates four alternative splicing patterns regarding novel exon 9A in mice. Grey boxes denote known exons (which use splice donor “GT_E9_” and acceptor “AG_E10_” sites), and red boxes mark the three new variants of alternative exon 9A (depending on use of AG_9A2-4_ and corresponding to predicted GlyR β-X2-4 protein variants). Note that the splice donor site only exists in mouse and bushbaby, whereas in all other investigated species this site is disrupted due to G/T substitution (“(T)”, see S3 Fig for sequence details). Also note that the splice acceptor site of E9A-3 is disrupted in rat due to additional G/A substitution (“(A)”). (B) Relative frequency of the four splicing patterns in embryonic (E17, open bars) and adult (P84-112, filled bars) mouse cerebral cortex. Apparently, splicing of exons 9A2-4 is not developmentally regulated. However, splicing of E9A-3 is significantly stronger compared to expression of mRNA with E9A-2 or E9A-4 splice variants. The numbers in brackets indicate the number of RNA sequencing replicates. (C) The donor site of the novel exon 9A (GT_E9A_) is as potent as that of the upstream exon (GT_E9_). The acceptor sites of the novel exon 9A (AG_E9A-2_, AG_E9A-3_ and AG_E9A-4_) are much weaker than that of the downstream exon 10 (AG_E10_), and the acceptor site of the most prevalent splice variant X3 is the weakest of the new acceptor sites. This suggests that inclusion of exon 9A is first defined by its strong donor site followed by selecting one of the three acceptor sites, with the preference of the AG_E9A-3_ acceptor site which is in nearest distance to the alternative splice donor site.

In mice, exon 9A is framed by a possible splice donor site “GT” and three predicted splice acceptor sites “AG” (named X2, X3, and X4 according to the three predicted splice variants listed in the NCBI nucleotide collection: accession numbers XM_006501004.1, XM_006501005.1, XM_006501006.1; Fig 1D). Hence, depending on the use of the different splice acceptor sites exons 9A-2, 9A-3, and 9A-4 are possibly expressed (Fig 2A). To determine the relative expression levels of exons 9A-2-4, we performed ontogenetic analyses of RNA sequencing data derived from embryonic (E17) and adult (P84-112) mouse cerebral cortex (NCBI GEO accession No. GSE39866) [29]. According to the number of reads the relative frequencies of mRNAs with exons 9A-2, 9A-3, and 9A-4 were determined (Fig 2B). Exon 9A-3 coding for ITYQLDGWPTDTLTTQ was predominantly expressed, representing 23.0 ± 2.1% (E17, E9A-3) and 19.5 ± 2.6% (P84-112, E9A-3) of GlyR β-coding mRNA (Fig 2B). This finding raised the question as to why E9A-3 expression is significantly higher compared to expression of E9A-2 or E9A-4. Therefore, we again performed computational analyses using the maximum entropy modeling of short sequence motifs [28]. The results show that the donor site of exon 9A (GT_E9A_) is as potent as that of the constitutive upstream exon (GT_E9_), whereas the acceptor sites of the E9A-2-4 (AG_E9A-2_, AG_E9A-3_ and AG_E9A-4_) are much weaker than that of the constitutive downstream exon (AG_E10_). This finding suggests that splicing of E9A is first defined by its strong donor site GT_E9A_ followed by preferential selection of the nearest splice acceptor site AG_E9A-3_.

**Fig 3:**
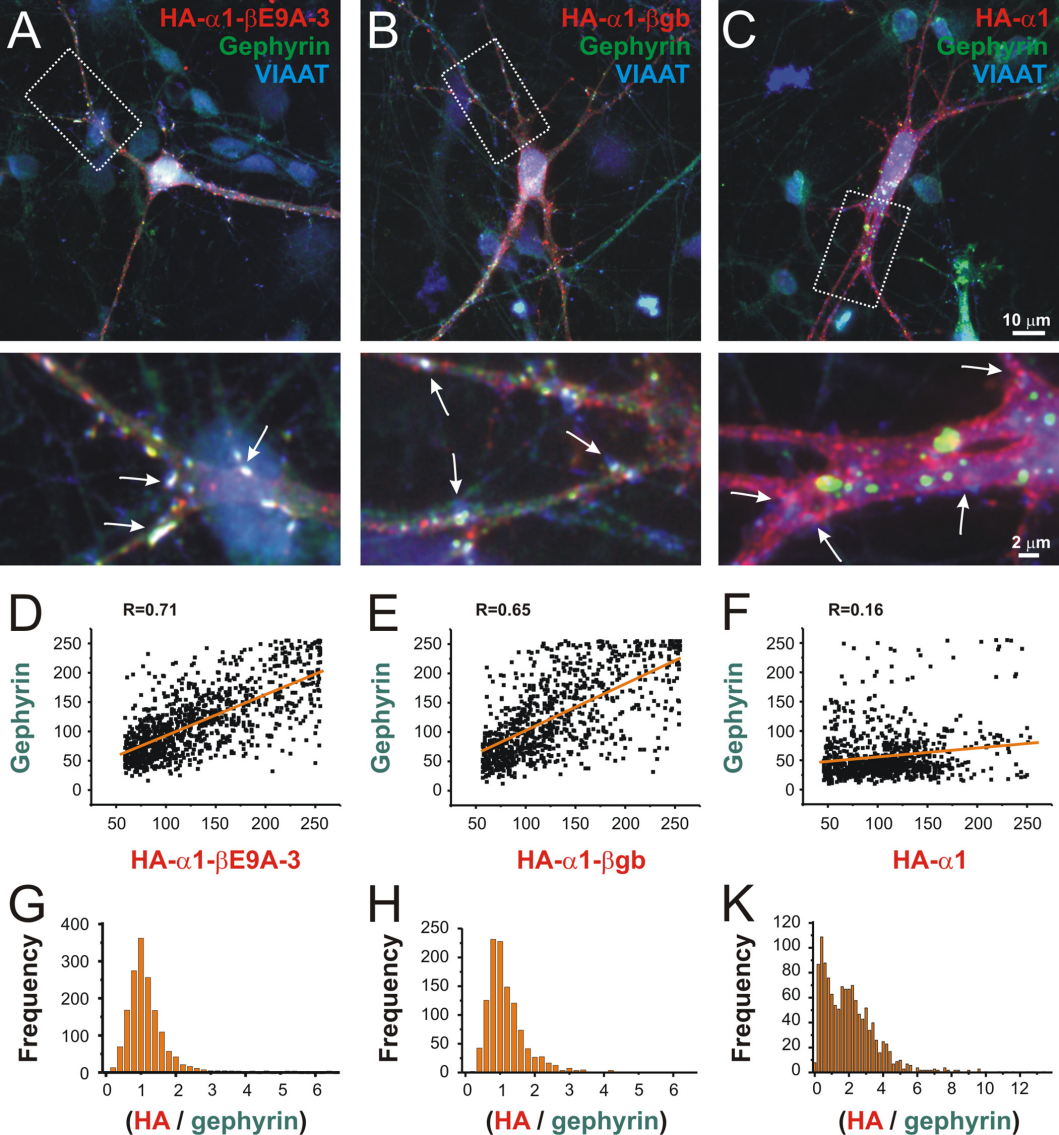
The exon 9A-3-coding sequence facilitates postsynaptic GlyR localization in the somatodendritic compartment of primary spinal cord neurons. (A-C) Spinal cord neurons were transfected with plasmids coding for the chimeric GlyR α1 with the new GlyR β insert (A, HA-α1-βE9A-3), the established gephyrin-binding variant (B, HA-α1-βgb), or without any additional sequence (C, HA-α1). (D-F) Quantitative analysis of postsynaptic GlyR α1-βE9A-3 (HA-signal peaks) and postsynaptic gephyrin revealed strong positive correlation of fluorescence intensities (R = 0.71, D). The established gephyrin-binding GlyR α1-βgb produced comparable results (R = 0.65, E). In contrast, postsynaptic gephyrin-associated clustering of GlyR α1 without any additional sequence in the large cytosolic loop was not evident (R = 0.16, F). (G-K) Histograms show the distributions of postsynaptic HA-signals in relation to locally corresponding gephyrin fluorescence intensities (HA / gephyrin fluorescence intensity ratio, bin size 0.2). Note that a value of 1 indicates perfect overlap. Note the different scale of the X-axis in panel K, and that values are mostly below or above the value of 1 in the case of gephyrin-independent postsynaptic clustering of GlyR HA-α1. Scale bars: 10 μm and 2 μm.

To characterize the functional impact of E9A-3 on neuronal GlyR expression, we generated chimeric GlyR constructs with the E9A-3-coding sequence ITYQLDGWPTDTLTTQ in the large cytosolic loop between transmembrane domains 3 and 4 of the α1 subunit (Fig 3A, “HA-α1-βE9A-3”). This is an established approach which allows investigation of short peptides with regard to synaptic receptor targeting because α1-GlyRs do not contain synaptic trafficking signals [5,7]. We visualized synaptic targeting and co-localization of the GlyR HA-α1-βE9A-3 chimera with postsynaptic gephyrin in primary spinal cord neurons using rat monoclonal anti-HA antibody (clone 3F10), guinea pig polyclonal antibody against the vesicular inhibitory neurotransmitter transporter VIAAT, and rabbit polyclonal anti-gephyrin antibody (Fig 3A). GlyR HA-α1-βE9A-3 formed postsynaptic receptor clusters which co-localized as well as the established gephyrin-binding chimera GlyR HA-α1-βgb [5] with postsynaptic gephyrin in the somatodendritic compartment of transfected neurons (Fig 3A and 3B; see S4 Fig for grey scale images). The chimera GlyR HA-α1-βgb harbors the gephyrin binding sequence RSNDFSIVGSLPRDFELS ^6^, was originally used to study gephyrin-dependent postsynaptic receptor dynamics [5] and therefore represents a suitable positive control. On the other hand, as expected, GlyR HA-α1 without any insert did not show remarkable co-localization with postsynaptic gephyrin (Fig 3C). Quantitative analysis of postsynaptic receptor localization at gephyrin-positive synapses was performed as described [7] and showed that GlyR HA-α1-βE9A-3 and GlyR HA-α1-βgb indeed performed equally well with regard to synaptic clustering (Fig 3D and 3E). In detail, HA-signal peaks and postsynaptic gephyrin fluorescence intensities were strongly positively correlated (HA-α1-βE9A-3: R = 0.71, HA-α1-βgb: R = 0.65), with both chimaeras exhibiting a high degree of congruency with locally corresponding gephyrin signals (Fig 3G and 3H, respectively), whereas correlation of the negative control HA-α1 with postsynaptic gephyrin signals was not evident (Fig 3F, R = 0.16) and hence congruency was rather poor (Fig 3K). Therefore, the βE9A-3 insert ITYQLDGWPTDTLTTQ can functionally substitute for the established βgb-gephyrin binding sequence RSNDFSIVGSLPRDFELS with regard to synaptic GlyR trafficking.

**Fig 4:**
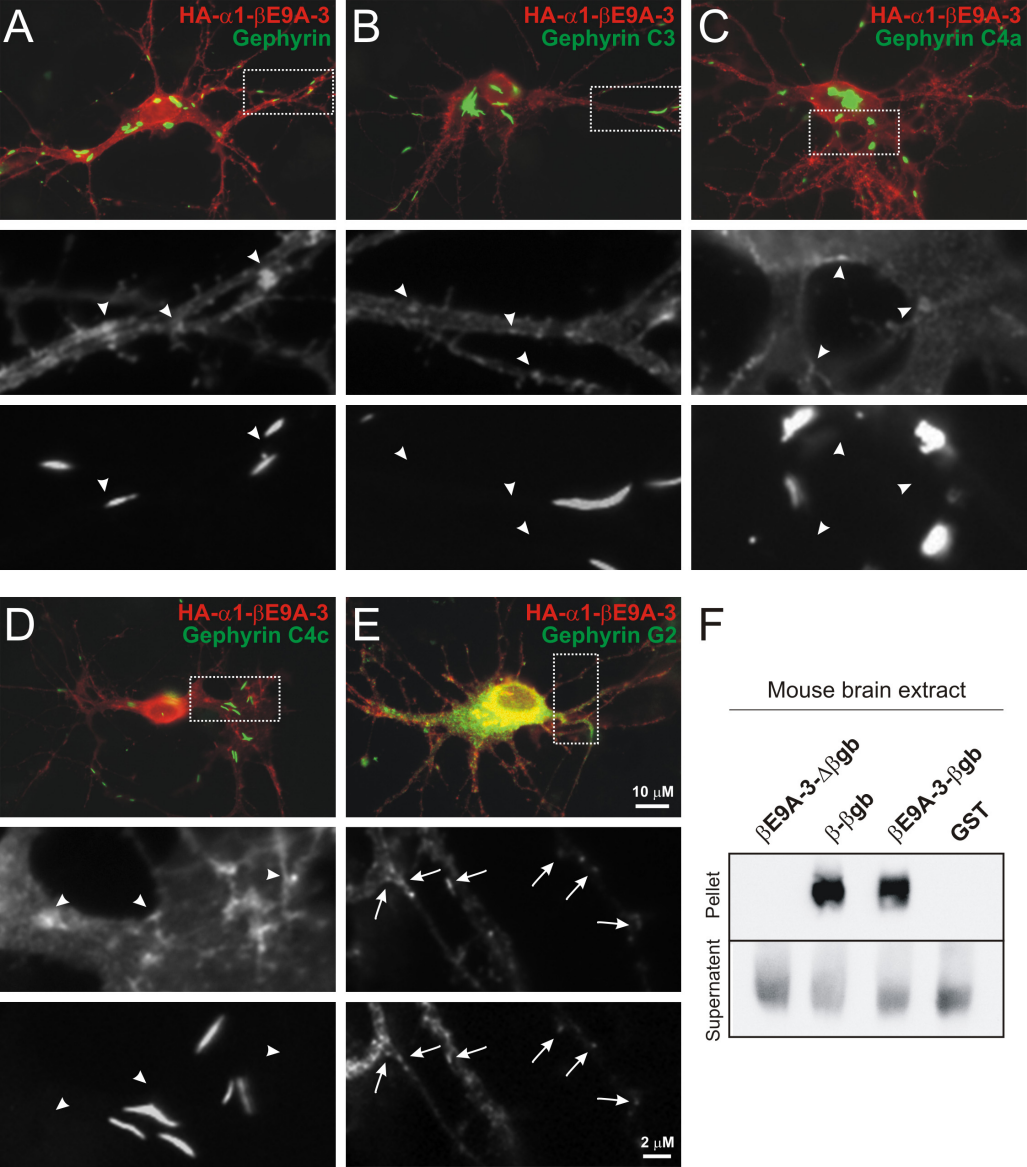
Gephyrin splice variant-specific recruitment of GlyR α1-βE9A-3. (A-E) Images of transfected primary hippocampal neurons show HA-α1-βE9A-3 and EGFP-tagged gephyrin splice variants. (A-D) No or apparently occasional overlap between HA-α1-βE9A-3 clusters and large aggregates of the over-expressed denoted different gephyrin splice variants was observed (arrowheads in grey scale high-power views). (E) In contrast, GlyR HA-α1-βE9A-3 co-localized with the G2-gephyrin splice variant (arrows). Scale bars, 10 μm and 2 μm. (F) GST co-sedimentation of gephyrin with the indicated large cytosolic GlyR loops between transmembrane domains 3 and 4 out of 350 μg mouse brain extract. GST alone was used as control. The western blot was probed with the monoclonal mAb3B11 antibody. Note that the GlyR βE9A-3-Δβgb loop was not detected in the pellet fraction, whereas GlyR β-βgb and βE9A-3-βgb loops co-sedimented with gephyrin.

Effective postsynaptic targeting of HA-α1-βE9A-3 to gephyrin-positive synapses suggests that the splice insert encoded by E9A-3 interacts with gephyrin, as does the βgb-sequence in the GlyR HA-α1-βgb chimera [5]. To address this possibility, we first performed an immunochemical analysis of transfected primary hippocampal neurons expressing GlyR HA-α1-βE9A-3 and different EGFP-tagged gephyrin splice variants (Fig 4A–4E), as described recently [7]. Surprisingly, HA-α1-βE9A-3 did not show co-localization with the different gephyrin splice variants C3, C4a, or C4c (Fig A-D; for the revised gephyrin nomenclature see ^22^), but HA-α1-βE9A-3 apparently co-clustered with G2-gephyrin (Fig 4E) [21,30], a splice variant which is known to be involved in regulation of postsynaptic gephyrin dynamics in spinal cord and hippocampal neurons [12,13]. These results suggest that the E9A-3 encoded sequence specifically interacts with the G2-gephyrin splice variant [21]. To investigate this possibility, we performed co-sedimentation assays using the large cytosolic loop between transmembrane domains 3 and 4 of the different GlyR β subunit variants as baits (Fig 4F). Surprisingly, the GST-tagged loop with βE9A-3 but without the βgb-gephyrin binding sequence (βE9A-3-Δβgb) did not co-sediment with gephyrin when Western blots were probed with the mAb3B11 antibody (Fig 4F), whereas GST-tagged loops with the βgb-gephyrin binding sequence motif (β-βgb and βE9A-3-βgb) co-sedimented with full-length gephyrin (Fig 4F). These results show that the E9A-3 encoded peptide ITYQLDGWPTDTLTTQ does not directly interact with gephyrin, and they also reveal that βE9A-3 does not interfere with βgb-dependent direct gephyrin interaction. Thus, ITYQLDGWPTDTLTTQ encoded by E9A-3 confers specific association of GlyRs with the G2-gephyrin splice variant [21] without necessarily interacting directly with gephyrin.

## Discussion

GlyRs are involved in synaptic neuronal communication and play an important role as they not only counterbalance excitation but also contribute to the spatiotemporal coordination of synaptic integration that is relevant for behavior and motor coordination [3,31]. Emerging evidence supports a decisive role of alternative RNA splicing in the functional diversification of gene products involved in inhibitory neuronal communication in health and disease [3,11–16,32,33]. Here, we identify a genomic sequence variation that gives rise to species-specific splice donor sites and enables functional diversification of the inhibitory GlyR; the results demonstrated that GlyRs with the βE9A-3 insert preferentially target inhibitory synapses which contain the G2-gephyrin splice variant as postsynaptic scaffold and receptor clustering protein. Furthermore, our study identifies the *Glrb* gene as new hot-spot of evolutionary diversification of mammalian protein function which is relevant for phylogenetic analysis.

Computational analysis of the RNA sequence database of mouse cerebral cortex [29] evidenced mRNA expression of predicted GlyR β splice variants (X2-4). However, expression of the GlyR βE9A-3 (X3) splice variant prevails with 20% of GlyR β-coding mRNA in embryonic and adult mouse cerebral cortex, compared to only ~7% for βE9A-2 (X2) or βE9A-4 (X4) expression. This is an unusual finding given the lowest predicted strength of the splice acceptor site that gives rise to GlyR βE9A-3 expression. Thus, the splice machinery seems to prefer the nearest splice acceptor site. Our analyses furthermore provide evidence for constitutive expression of the new GlyR β splice variant as the relative expression levels were comparable among different regions of the central nervous system (Fig 1A), at different developmental stages (Fig 2B and S1 Fig and NCBI GEO accession No. GSE39866, data not shown), in the aging hippocampus (NCBI GEO accession No. GSE61915, data not shown), or in disease conditions (S2 Fig). Actually, ~20% of GlyR β-coding mRNA contains the new splice insert irrespectively of whether striatum of Huntington mice [34] or hippocampus of epileptic mice [35] were analyzed (S2 Fig). Consistently, mice with genetically determined neural network hypo- and hyperexcitability [3] did not show alterations in the relative expression level of GlyR βE9A (S1 Fig). Finally, the relative abundance of E9A-3 remained invariant in acute/subacute phases of a mouse model of contusive spinal cord injury (NCBI GEO accession No. GSE45376, data not shown). Hence, expression of GlyR βE9A is constitutive in mice, which can actually be expected from a genomic hot spot that produced an intermediate step of evolutionary diversification of protein function.

The correlation of postsynaptic GlyR α1-βE9A-3 and neuronal endogenous gephyrin distributions in neurons revealed a high degree of congruency of both proteins at VIAAT-positive glycinergic/GABAergic synapses in the somatodendritic compartment. Hence, the splice insert encoded by E9A-3 is sufficient to convey synaptic GlyR trafficking independently of the well-known βgb-gephyrin binding sequence motif in the GlyR β subunit [5,6]. The results of the immunochemical analysis of transfected neurons furthermore identified a selective association of the GlyRs with the βE9A-3 splice insert with the G2-gephyrin splice variant. However, the co-sedimentation assays do not support a direct interaction of the GlyR βE9A-3 splice insert with gephyrin, which suggests that an intermediate yet unknown protein is required for its association with G2-gephyrin. As postsynaptic GlyR α1-βE9A-3 clustering was observed at virtually all gephyrin-positive synapses in transfected primary neurons, the G2-gephyrin splice variant seems to be basically expressed at inhibitory synapses. The G2-gephyrin splice variant was shown to displace both gephyrin and GlyR from postsynaptic densities [13], hence “solubilizes” the postsynaptic gephyrin matrix, suggesting that species-specific expression of GlyRs with the βE9A-3 insert and its preference for association with the G2-gephyrin splice variant could represent an evolutionary attempt to establish a novel synapse “plasticizer” mechanism that would add another degree of freedom to inhibitory synaptic plasticity [36].

**Fig 5:**
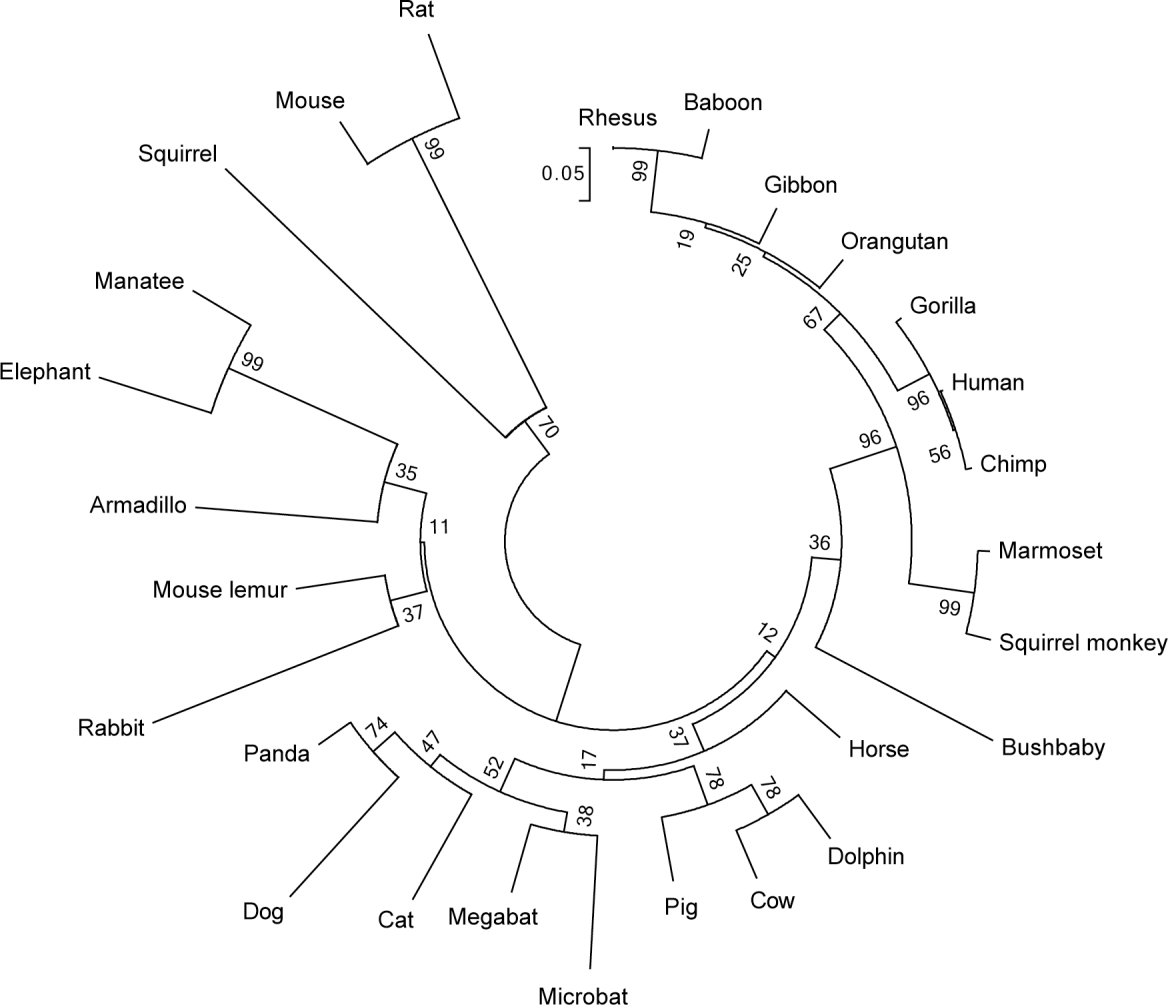
Neighbor-joining phylogenetic tree of genomic sequences containing GlyR βE9A. The optimal tree with sum of branch length of 3.424 is shown. The tree is drawn to scale, with branch lengths in the same units as those of the evolutionary distances used to infer the phylogenetic tree. The sequences in rodents evolved much faster than that in primates. Interestingly, although squirrel grouped together with mouse and rat as an outlier, the splicing donor site of GlyR βE9A is deleted in squirrel.

In addition to these novel functional data our study is useful in bridging the sequences of higher primates to close non-primates. In fact, computational analysis of the species-specific sequence variations in the *Glrb* gene region revealed differences (Fig 5) to the recently proposed phylogenetic tree of *Eutherian* evolution [37]. Based on the evolutionary history of the GlyR βE9A sequences that was inferred using the Neighbor-Joining method, rodents and primates may not be as closely related as originally thought, bushbaby seems not as closely related to primates but may have evolved much earlier, elephant and manatee may have evolved much later (not from a common *Eutherian* ancestor but rather from an ancestor of armadillo), and the phylogenetic tree of the *Glrb* gene region sets mouse and rat apart from rabbit. However, whether the thymine-to-guanine sequence variation in *Glrb* occurred independently in bushbaby and mouse, or whether the species variation evolved much earlier in a common ancestor of both species but subsequently disappeared in all *Eutherian*-descending species except bushbaby and mouse remains an open question.

## Conclusions

Our study identified a new genomic hot spot region that takes a snapshot of evolutionary diversification of protein function and adds to our understanding of phylogenetic relationships.

## Supporting Information

S1 Fig Changes in neural network homeostasis do not influence GlyR β E9A splicing. The agarose gel does not reveal apparent changes of the relative amount of GlyR β with E9A (arrow) compared to the band that corresponds to *Glrb* transcripts without E9A (arrowhead). Note that, as described recently [3], *Hprt* ^α3L185L+/0^;*Pvalb* ^Cre+/-^ and *Hprt* ^α3L185L+/0^;*Camk2a* ^Cre+/-^ mice are characterized by decreased and increased neural network excitability, respectively.
(TIF)

S2 Fig Chronic neurological disorders are not associated with changes of GlyR β exon 9A splicing. The agarose gel does not reveal apparent changes of the relative amount of GlyR β with E9A (arrow) compared to the band that corresponds to *Glrb* transcripts without E9A (arrowhead). Amplification of cDNA probes derived from the intrahippocampal kainate model of epilepsy in mice is shown left-hand. “Ipsilateral” designates the injected hippocampus of two animals (#1, #2), while “contralateral” corresponds to probes derived from the contralateral hippocampi of the two injected animals. Note that the epileptic focus was located in the dorsal ipsilateral hippocampus, where kainate was injected, and that dorsal and ventral hippocampi were collected separately. We also analyzed GlyR β exon 9A splicing in the striatum of animals with Huntington’s disease (right-hand). Again, no difference between control (C57BL/6) and Huntington mice was detected.
(TIF)

S3 Fig Alignment of genomic sequences corresponding to E9A-3. Note that the reverse complement sequence is shown. Sequence regions corresponding to splice donor and acceptor sites in the mouse genome are boxed. For direct access to annotated databases and sequences see following hyperlinks: Mouse, Dog, Horse, Cat, Cow, Rhesus, Orangutan, Chimp, Marmoset, Rat
(TIF)

S4 Fig The E9A-3-coding sequence facilitates postsynaptic GlyR localization in spinal cord neurons. (A-C) Grey scale images of the high-power views of merged fluorescent signals in Fig 3 are shown.
(TIF)
