# Supplementary figures and images for: Identification of a New Genomic Hot Spot of Evolutionary Diversification of Protein Function
Source: PLoS One. 2015 May 8;10(5):e0125413. doi: 10.1371/journal.pone.0125413 (PMC4425505; doi:10.1371/journal.pone.0125413)

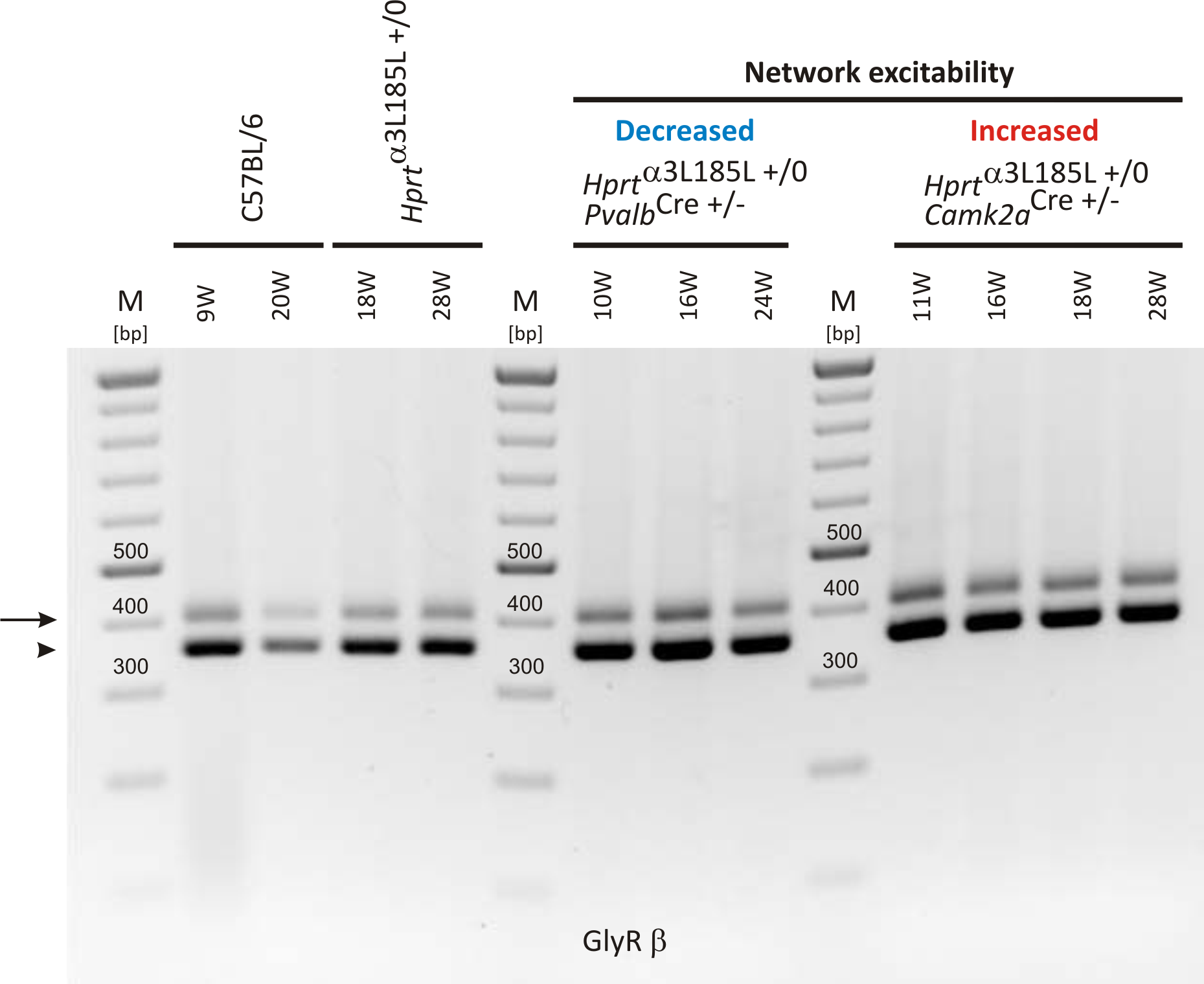

Supplement: S1 Fig — The agarose gel does not reveal apparent changes of the relative amount of GlyR β with E9A (arrow) compared to the band that corresponds to Glrb transcripts without E9A (arrowhead). Note that, as described recently [3], Hprt α3L185L+/0;Pvalb Cre+/- and Hprt α3L185L+/0;Camk2a Cre+/- mice are characterized by decreased and increased neural network excitability, respectively. (TIF) [file pone.0125413.s001.tif]

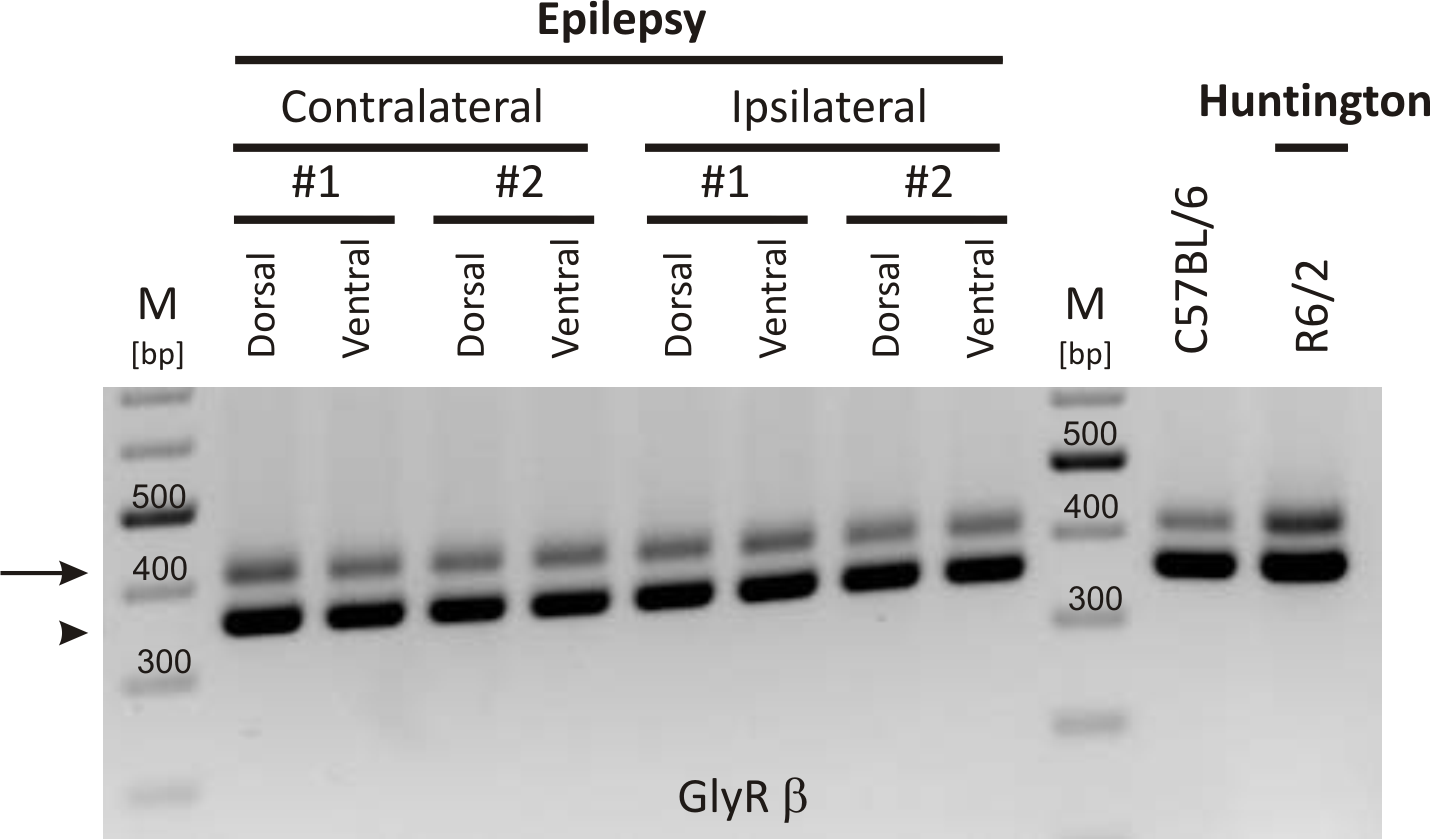

Supplement: S2 Fig — The agarose gel does not reveal apparent changes of the relative amount of GlyR β with E9A (arrow) compared to the band that corresponds to Glrb transcripts without E9A (arrowhead). Amplification of cDNA probes derived from the intrahippocampal kainate model of epilepsy in mice is shown left-hand. “Ipsilateral” designates the injected hippocampus of two animals (#1, #2), while “contralateral” corresponds to probes derived from the contralateral hippocampi of the two injected animals. Note that the epileptic focus was located in the dorsal ipsilateral hippocampus, where kainate was injected, and that dorsal and ventral hippocampi were collected separately. We also analyzed GlyR β exon 9A splicing in the striatum of animals with Huntington’s disease (right-hand). Again, no difference between control (C57BL/6) and Huntington mice was detected. (TIF) [file pone.0125413.s002.tif]

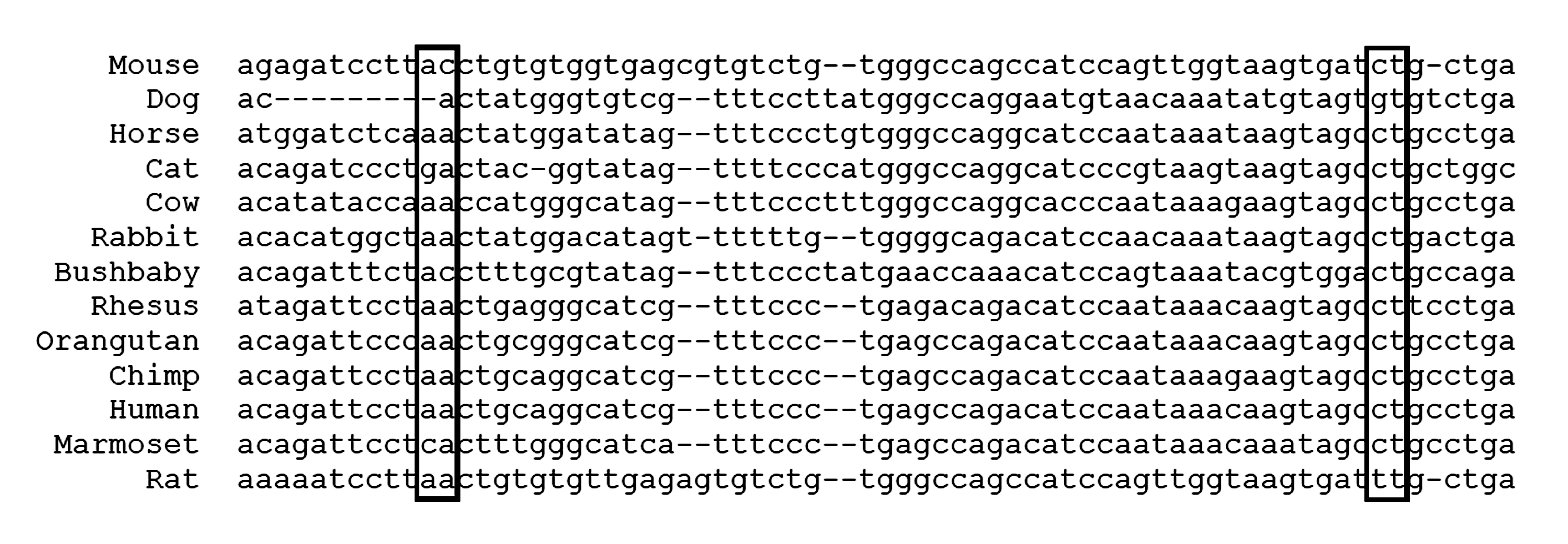

Supplement: S3 Fig — Note that the reverse complement sequence is shown. Sequence regions corresponding to splice donor and acceptor sites in the mouse genome are boxed. For direct access to annotated databases and sequences see following hyperlinks: Mouse, Dog, Horse, Cat, Cow, Rhesus, Orangutan, Chimp, Marmoset, Rat (TIF) [file pone.0125413.s003.tif]

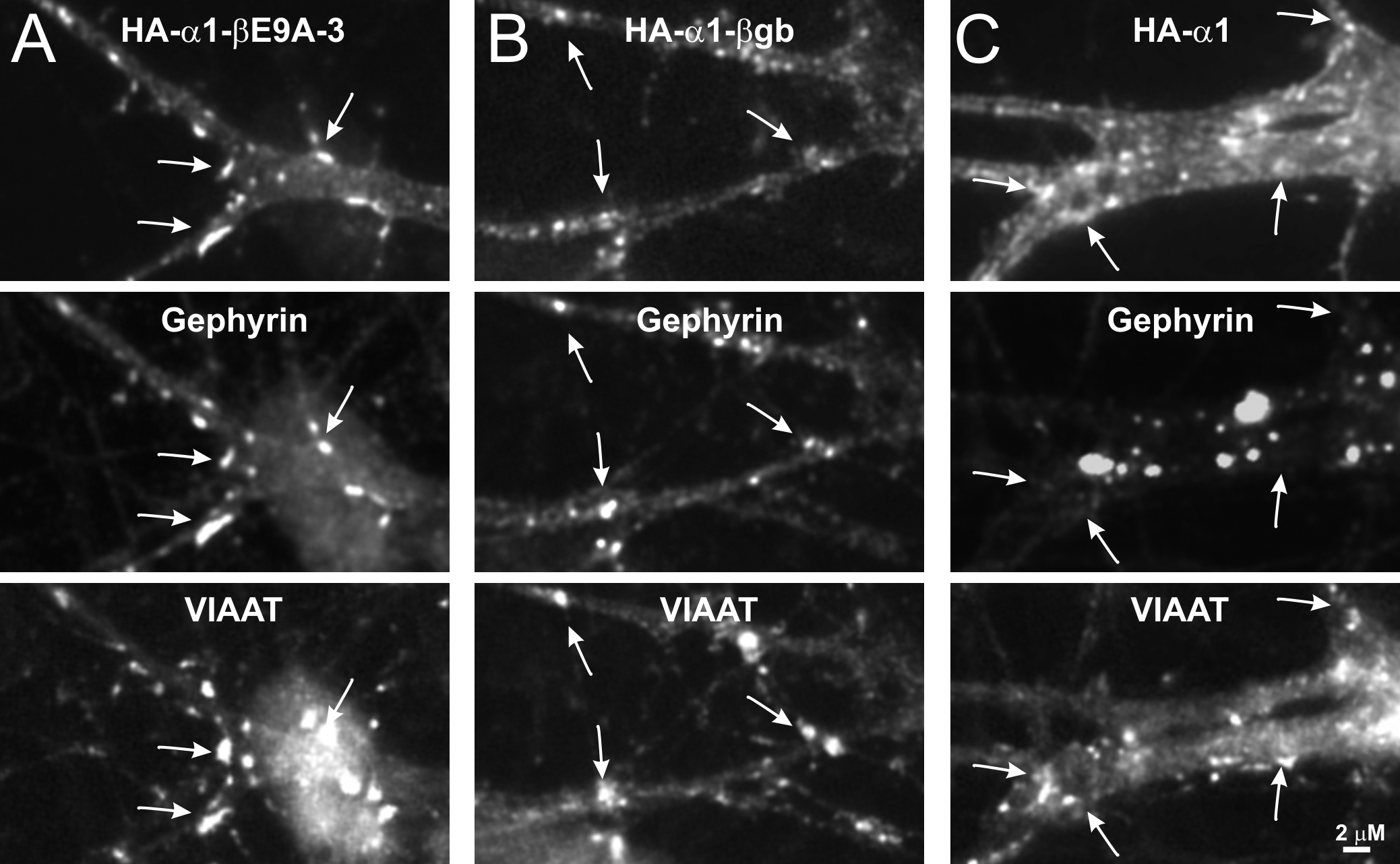

Supplement: S4 Fig — (A-C) Grey scale images of the high-power views of merged fluorescent signals in Fig 3 are shown. (TIF) [file pone.0125413.s004.tif]
